# Cyclin B1/Cdk1 Phosphorylation of Mitochondrial p53 Induces Anti-Apoptotic Response

**DOI:** 10.1371/journal.pone.0012341

**Published:** 2010-08-23

**Authors:** Danupon Nantajit, Ming Fan, Nadire Duru, Yunfei Wen, John C. Reed, Jian Jian Li

**Affiliations:** 1 Department of Radiation Oncology, University of California Davis, Sacramento, California, United States of America; 2 Program of Cell Death and Apoptosis, Sanford-Burnham Medical Research Institute, La Jolla, California, United States of America; 3 Cancer Center, University of California Davis, Sacramento, California, United States of America; Roswell Park Cancer Institute, United States of America

## Abstract

The pro-apoptotic function of p53 has been well defined in preventing genomic instability and cell transformation. However, the intriguing fact that p53 contributes to a pro-survival advantage of tumor cells under DNA damage conditions raises a critical question in radiation therapy for the 50% human cancers with intact p53 function. Herein, we reveal an anti-apoptotic role of mitochondrial p53 regulated by the cell cycle complex cyclin B1/Cdk1 in irradiated human colon cancer HCT116 cells with p53^+/+^ status. Steady-state levels of p53 and cyclin B1/Cdk1 were identified in the mitochondria of many human and mouse cells, and their mitochondrial influx was significantly enhanced by radiation. The mitochondrial kinase activity of cyclin B1/Cdk1 was found to specifically phosphorylate p53 at Ser-315 residue, leading to enhanced mitochondrial ATP production and reduced mitochondrial apoptosis. The improved mitochondrial function can be blocked by transfection of mutant p53 Ser-315-Ala, or by siRNA knockdown of cyclin B1 and Cdk1 genes. Enforced translocation of cyclin B1 and Cdk1 into mitochondria with a mitochondrial-targeting-peptide increased levels of Ser-315 phosphorylation on mitochondrial p53, improved ATP production and decreased apoptosis by sequestering p53 from binding to Bcl-2 and Bcl-xL. Furthermore, reconstitution of wild-type p53 in p53-deficient HCT116 p53^−/−^ cells resulted in an increased mitochondrial ATP production and suppression of apoptosis. Such phenomena were absent in the p53-deficient HCT116 p53^−/−^ cells reconstituted with the mutant p53. These results demonstrate a unique anti-apoptotic function of mitochondrial p53 regulated by cyclin B1/Cdk1-mediated Ser-315 phosphorylation in p53-wild-type tumor cells, which may provide insights for improving the efficacy of anti-cancer therapy, especially for tumors that retain p53.

## Introduction

In mammalian cells, tumor suppressor p53 plays important roles in a diversity of physiologic functions. Cellular p53 functions as a tumor suppressor by increasing genomic stability and inhibiting cell transformation [1], initiating apoptosis upon defected DNA damage repair [2], [3]. During stress, p53 rapidly translocates to the outer-membrane of mitochondria and engages with Bcl-2 family proteins, leading to permeabilization of the mitochondrial outer-membrane, followed by the release of cytochrome c and initiation of apoptosis [4], [5], [6]. p53-regulated pro-apoptotic function is believed to contribute to the efficacy of anti-cancer therapy [7]. p53 may also possess cell survival activity, as suggested by the radioadaptive resistance of p53-positive cells treated with fractionated irradiation [8], and the observation that lost-of–function p53 has been linked with increased cell sensitivity to radiation and enhanced apoptosis [9], [10]. Mitochondrial localization of p53 can exert an anti-apoptotic function [11], [12]. In addition, p53 reportedly acts as a pro-survival factor by promoting mitochondrial biogenesis [13], mitochondrial DNA repair and synthesis [14], [15], [16], and respiration [17]. The molecular mechanism underlying such p53-mediated anti-apoptotic response in the context of genotoxic stress remains to be elucidated.

One cause of p53 activation by radiation is due to its phosphorylation by the cell cycle regulator cyclin B1 and its kinase partner Cdk1 [18]. The cyclin B1/Cdk1 complex (so-called Mitosis Promoting Factor, MPF) controls the mitotic entrance from G_2_ to M phase [19], [20]. As a checkpoint, the cyclin B1/Cdk1 complex also arrests cell cycle at the G_2_/M phase allowing cells sufficient time to repair damaged DNA and influencing various pro-survival signaling pathways before entering mitosis [21], [22]. Elevated levels of cyclin B1/Cdk1 activity account for the chemo/radio-resistance in post-treatment to head and neck cancers [23], [24]. Aberrant activity of cyclin B1/Cdk1 is found in the radiation-derived tumor resistance [25], and inhibition of cyclin B1/Cdk1 activity enhances tumor radiosensitivity by increasing apoptosis [26], [27]. The cyclin B1/Cdk1 complex is able to interact with both pro- and anti-apoptotic proteins including BAD, Bcl-2, Bcl-xL, Mcl-1, caspase-9 and survivin [28], [29], [30], [31], [32]. However, the exact mechanisms of cyclin B1/Cdk1-mediated mitochondrial functions and their potential correlations with p53 are still unknown.

p53, as a pivotal factor in both gene expression [33] and cell cycle regulation [34], [35], can be potentially phosphorylated at least on 17 amino-acid sites by various kinases accounting for its diversified roles. In particular, the Ser-315 residue on p53 is phosphorylated by cyclin B1/Cdk1 [36]. In this study, we examined cyclin B1/Cdk1-mediated p53 Ser-315 phosphorylation occurred in mitochondria and its relevance to the radioresistant phenotype of p53 wild-type tumor cells. Our data demonstrate a novel pro-survival signaling network initiated by radiation-induced mitochondrial targeting of cyclin B1, Cdk1 and p53, and subsequent mitochondrial p53 Ser-315 phosphorylation causing enhanced mitochondrial ATP generation and mitochondrial membrane potential. These results suggest that DNA damaging anti-cancer reagents can activate an adaptive response via nuclear-to-mitochondrial protein trafficking to protect mitochondrial integrity and suppress apoptosis.

## Results

### Enrichment of mitochondrial p53, cyclin B1 and Cdk1 by irradiation

**Figure 1:**
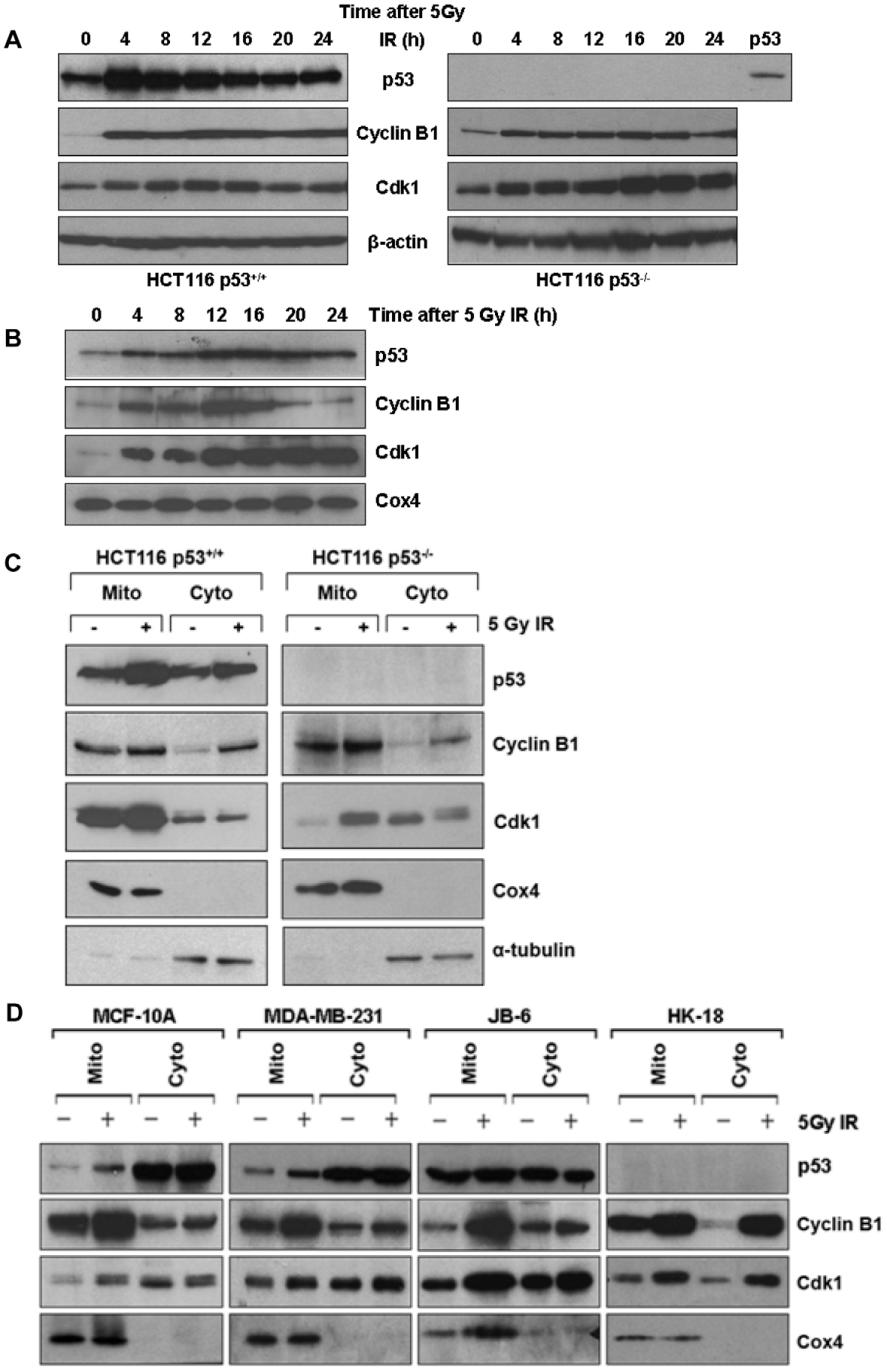
Mitochondrial localization of p53, cyclin B1 and Cdk1 was enhanced by irradiation. Human colon cancer HCT116 cells with wild-type p53^+/+^ (left panel) or p53^−/−^ deficient (right panel) were treated by 5 Gy radiation. (**A**) Induction of p53, cyclin B1 and Cdk1 in whole cell lysate were determined by immunoblotting. (**B**) Mitochondrial p53, cyclin B1 and Cdk1 were measured by immunoblotting with 20 µg total protein of mitochondrial fraction purified from control and irradiated HCT116 p53^+/+^ cells (Cox4 as mitochondrial fraction marker). (**C**) Mitochondrial (Mito) and cytosolic (Cyto) p53, cyclin B1 and Cdk1 were detected by immunoblotting with 20 µg total protein of each fraction purified from irradiated HCT116 p53^+/+^ and p53^−/−^ cells (Cox4 and α-tubulin served as mitochondrial and cytosolic markers respectively). (**D**) Mitochondrial p53, cyclin B1 and Cdk1 were detected by immunoblotting with 20 µg total protein of mitochondrial fraction purified from irradiated and control human breast epithelial cell MCF-10A, breast cancer cell MDA-MB-231, immortalized normal mouse skin epithelial JB6 cells and immortalized human skin keratinocytes (HK18) with deficient p53.

Activation of p53 and cell cycle regulators has been well demonstrated in radiation-induced DNA damage response [37]. Induction of p53, cyclin B1 and Cdk1 was confirmed in irradiated human colon cancer cell with wild-type p53 (HCT116 p53^+/+^) compared with the p53 deficient (HCT116 p53^−/−^) cells. The steady-state levels of p53, cyclin B1 and Cdk1 were increased in HCT116 p53^+/+^ (Figure 1A, left panel) and the induction of cyclin B1 and Cdk1 was also observed in the HCT116 p53^−/−^ cells following exposure to radiation (Figure 1A, right panel), indicating that radiation-induced cyclin B1/Cdk1 expression was independent of p53 status. Concomitantly, a basal level of p53, cyclin B1 and Cdk1 was confirmed in the mitochondria of HCT116 p53^+/+^ cells and a substantial amount of all three proteins was detected in the mitochondria of HCT116 p53^+/+^ cells after irradiation (Figure 1B). Compared to their cytosolic distribution, a relatively higher concentration of p53, cyclin B1 and Cdk1 was detected in mitochondria and the mitochondrial influx of all three proteins were significantly enhanced in HCT116 p53^+/+^ cells by irradiation (Figure 1C). In the p53 deficient HCT116 p53^−/−^ cells, a basal mitochondrial cyclin B1/Cdk1 was also present in the mitochondria and was enhanced by irradiation, indicating that mitochondrial influx of p53 and cyclin B1/Cdk1 was mutually independent. The mitochondrial enrichment of cyclin B1 and Cdk1 was also evidenced in other cell lines with both wild-type and mutant p53 such as MCF-10A, MDA-MB-231 and JB6, and p53 deficient HK-18 cells (Figure 1D). These data indicate that cyclin B1 and Cdk1 are present in the mitochondria of mammalian cells and their mitochondrial influx is significantly enhanced by DNA damage stress.

### Active mitochondrial Cdk1 interacted and phosphorylated p53 at Ser-315

**Figure 2:**
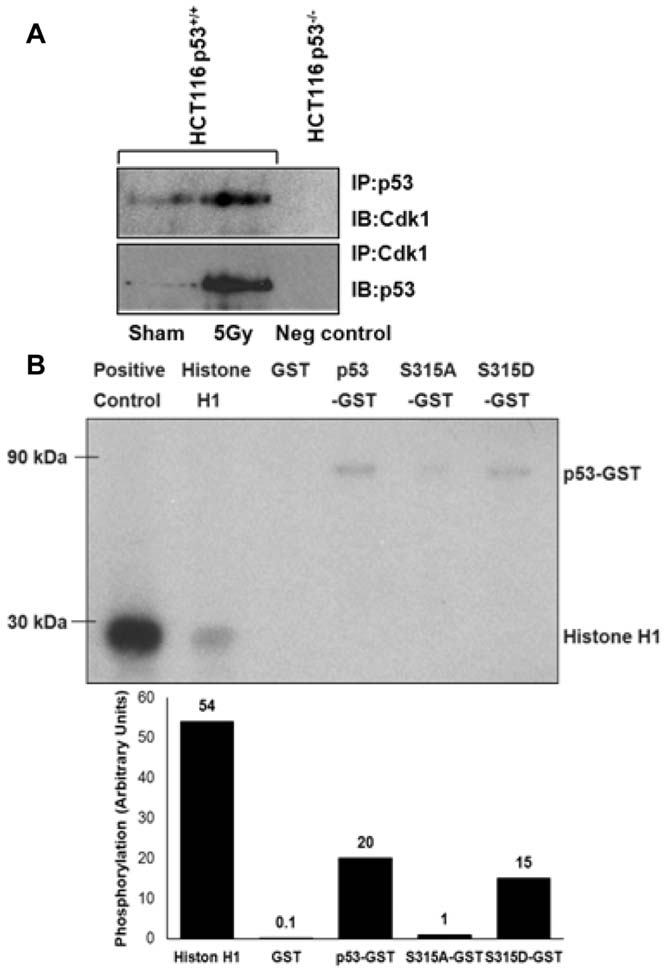
Mitochondrial Cdk1 interacted and phosphorylated p53. (**A**) Mitochondria fractions (100 µg) from control (sham-irradiated) and irradiated HCT116 p53^+/+^ cells (HCT116 p53^−/−^ cells included as a negative control) were immunoprecipitated (IP) with p53 antibody followed by immunoblotting (IB) with Cdk1 antibody (top panel); IP with Cdk1 antibody followed by IB with p53 antibody (bottom panel). (**B**) Kinase assay was performed with Cdk1 purified with IP from total 2.4 mg of mitochondrial fraction isolated from irradiated HCT116 p53^+/+^ and incubated with 8 µg of GST-p53 (wild-type or mutants at S315A or S315D). The same amount of histone H1 was included as a positive phosphorylation substrate for Cdk1, and GST-protein only served as a negative control (mitochondrial Cdk1 mediated p53 phosphorylation was estimated by densitometry in comparison to the phosphorylation level of histone H1).

To determine whether mitochondrial cyclin B1/Cdk1 complex is capable to phosphorylate p53 in mitochondria, mitochondrial fractionation were performed from irradiated HCT116 p53^+/+^ cells and interaction between mitochondrial Cdk1 and p53 was detected by co-immunoprecipitation. Robust Cdk1/p53 interaction was detected in the mitochondrial fraction of irradiated HCT 116 p53^+/+^ cells (Figure 2A); this increased Cdk1/p53 interaction started as early as 2 to 4 hours after irradiation (Figure S1A). To verify whether mitochondria-translocated Cdk1 is active, *in vivo* kinase assay was performed using immune-isolated Cdk1 from mitochondrial fraction of irradiated HCT 116 p53^+/+^ cells with GST-tagged synthesized p53 (wild-type, Ser-315 to Ala or Ser-315 to Asp mutants) and Histone protein (H1) as the target substrates. Results in Figure 2B demonstrated that mitochondrial Cdk1 indeed demonstrated its kinase activity and phosphorylated wild-type p53 and the positive control Histone H1; in contrast, Cdk1 mediated phosphorylation was markedly reduced in the mutant p53 (S315A, phosphorylation inhibit) but not the phosphorylation counterfeit mutant p53 (S315D) [38]. The substrate specificity of the kinase assay was confirmed in Figure S1B. Together, these results demonstrate that mitochondrial translocated Cdk1 retains its kinase activity and is able to phosphorylate p53 at Ser-315.

### Mitochondrial p53 Ser-315 was specifically phosphorylated by mitochondrial cyclin B1/Cdk1 after irradiation

**Figure 3:**
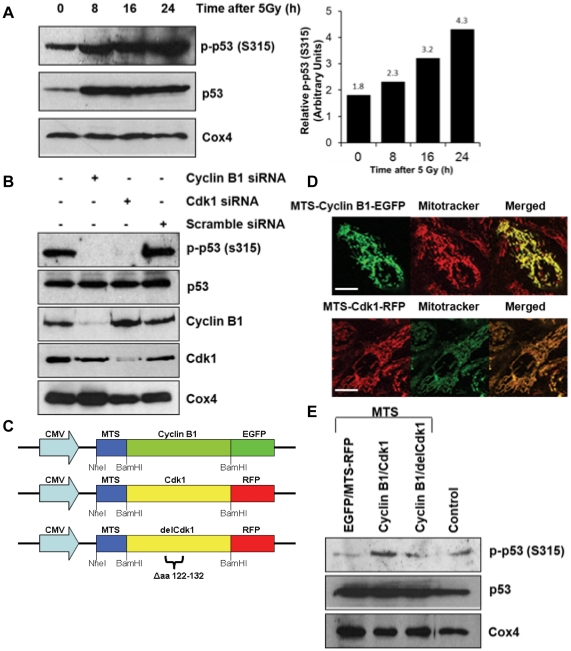
Radiation stimulated cyclin B1/Cdk1-mediated mitochondrial p53 Ser-315 phosphorylation. (**A**) p53 Ser-315 phosphorylation was detected by immunoblotting with anti-phospho-p53(S315) antibody with 20 µg mitochondrial fractions from irradiated HCT116 p53^+/+^. (**B**) Total protein and phosphorylated p53, cyclin B1, and Cdk1 were determined in irradiated HCT116 p53^+/+^ transfected with siRNAs against cyclin B1, Cdk1 or scrambled seq. (**C**) Structural illustration of fusion protein containing mitochondria-targeted-sequence (MTS) linked with cyclin B1-EGFP, Cdk1-RFP, as well as a mutant form delCdk1 with the deletion of catalytic domain of Cdk1 (aa 122–132). (**D**) Fluorescence images of HCT116 p53^+/+^ cells transfected with mitochondria-targeted cyclin B1-EGFP and Cdk1-RFP with Mitotracker mitochondria staining (scale bar = 5 µm). (**E**) Ser-315 phosphorylation of mitochondrial p53 was determined in HCT116 p53^+/+^ cells co-transfected with empty vectors MTS-EGFP/MTS-RFP, or with MTS-cyclin B1 fused with EGFP (MTS-cyclin B1) and MTS-Cdk1 fused with RFP (MTS-Cdk1), or with MTS-cyclin B1 fused with EGFP and MTS-delCdk1 fused with RFP (delCdk1). In addition, HCT116 p53^+/+^ cells without transfection was included as a control.

p53 Ser-315 phosphorylation has been shown to be induced by irradiation [39] and confirmed by our present study (Figure S2A). To address the question of whether the stress-induced p53 protein is, at least in part, translocated to mitochondria and its Ser-315 residue is targeted by cyclin B1/Cdk1 complex, we measured the levels of phosphorylated p53 in mitochondrial fractions isolated from HCT116 p53^+/+^ at different time intervals after irradiation. Although a high steady-level of radiation-induced total mitochondrial p53 was maintained throughout 24 hours after irradiation, phosphorylated p53 Ser-315 was markedly increased during the hours (Figure 3A), suggesting that the phosphorylation may happen within mitochondria after irradiation. The phosphorylation of mitochondrial p53 was further confirmed in irradiated mouse xenograft tissue as shown in Figure S2B. In agreement, inhibition of cyclin B1 or Cdk1 by short-interference siRNA knockdown drastically diminished the Ser-315 phosphorylation of mitochondrial p53 of irradiated HCT116 p53^+/+^ cells without altering the total mitochondrial p53 level (Figure 3B). Although the expression of cyclin B1 and Cdk1 was partially reduced in the whole cell lysate (Figure S2C), it is noteworthy that mitochondrial cyclin B1 and Cdk1 levels were markedly reduced by the specific siRNAs, suggesting that mitochondrial translocation of cyclin B1 and Cdk1 is sensitive to the overall cyclin B1 and Cdk1 cellular protein expression. In addition, siRNA knockdown of cyclin B1 did not affect mitochondrial translocation of Cdk1, and *vice versa*, indicating the independent mitochondrial influx of cyclin B1 and Cdk1 (Figure 3B).

To further understand the mitochondrial trafficking of cyclin B1 and Cdk1, we constructed vectors with mitochondria targeting sequence (MTS) derived from human cytochrome c oxidase subunit 8a (Cox8a) [40], [41] and inserted at the N-terminal of cyclin B1, Cdk1 and delCdk1 (truncated Cdk1 with deletion at its catalytic loop, residue 122–132, delCdk1) [42] as shown in Figure 3C. The specificity of MTS-mediated mitochondrial localization of cyclin B1 and Cdk1 was validated by western blot and fluorescence microscopy with Mitotracker (Figure 3D and Figures S2D and E). The mitochondrial p53 Ser-315 phosphorylation was significantly enhanced in cells co-transfected with MTS-cyclin B1 and MTS-Cdk1 (Figure 3E). In contrast, the mitochondrial p53 Ser-315 phosphorylation was partially compromised by co-transfecting MTS-cyclin B1 with MTS-delCdk1 that lacks the Cdk1 catalytic loop (Asp-128 and Lys-130 motif) for binding to γ-phosphate ATP required for the Cdk1-mediated phosphorylation [43]. Together, these results strongly suggest that mitochondrial p53 Ser-315 is specifically phosphorylated by mitochondrial cyclin B1/Cdk1 that was enhanced by the stress of ionizing radiation.

### p53 Ser-315 phosphorylation was involved in mitochondrial ATP production

**Figure 4:**
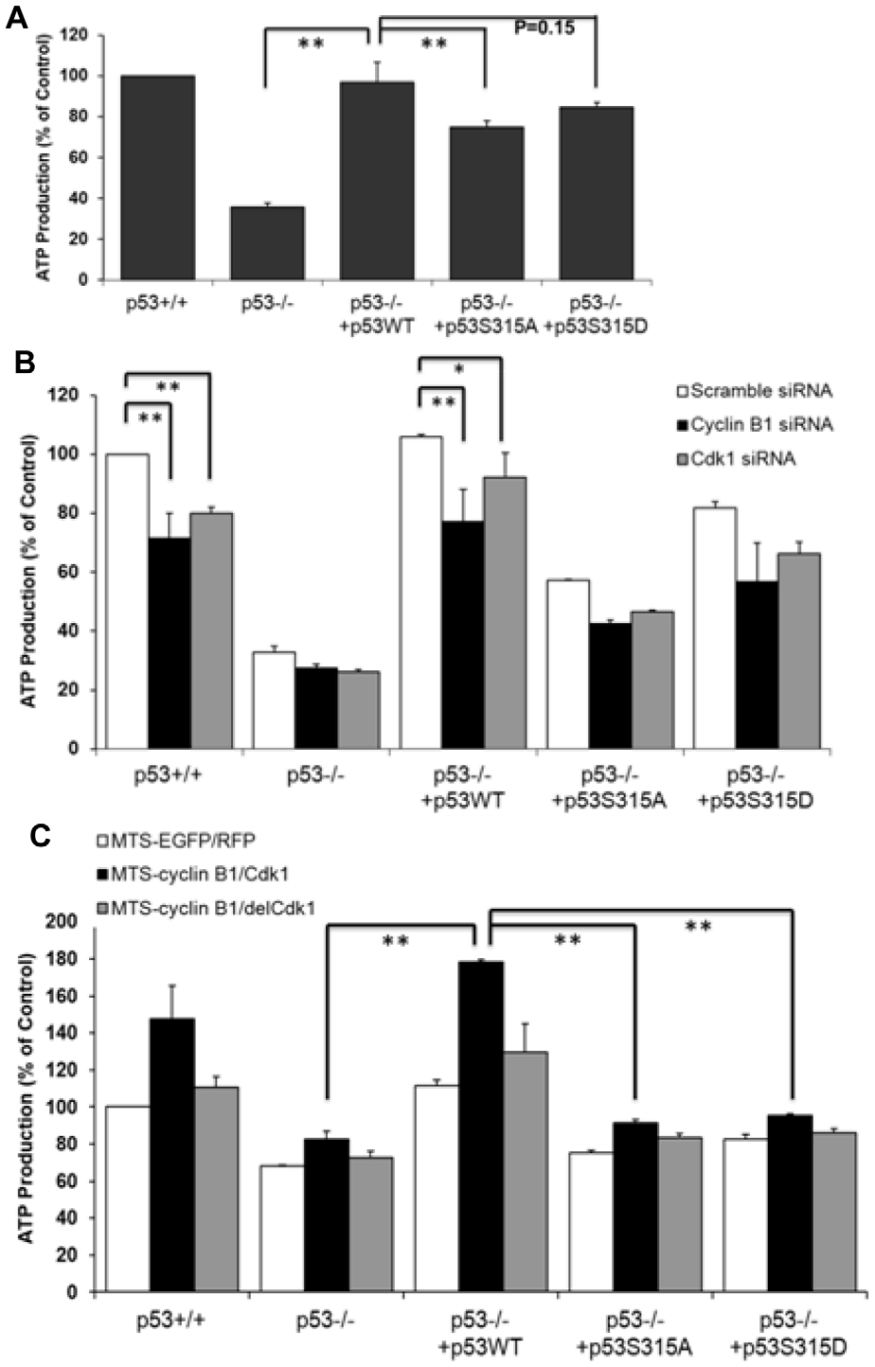
Cyclin B1/Cdk1-mediated p53 phosphorylation regulated mitochondrial ATP production. (**A**) Mitochondrial ATP production was measured in exponentially growing HCT116 p53^+/+^, HCT116 p53^−/−^, or stable HCT116 p53^−/−^ transfectants with p53 wild-type, or S315A/S315D mutants (n = 3; mean ± SEM; **P<0.01). (**B**) Mitochondrial ATP production was measured in the same set of cells additionally transfected with cyclin B1, Cdk1 or scrambled siRNAs for 24 h (n = 3; mean ± SEM; *P<0.05; **P<0.01). (**C**) Mitochondrial ATP production was measured in the same set of cells transfected with MTS-EGFP and MTS-RFP, or MTS-cyclin B1 and MTS-Cdk1, or MTS-cyclin B1 and MTS-delCdk1 (n = 3; mean ± SEM; **P<0.01).

To examine the function of the mitochondrial p53 phosphorylated by cyclin B1/Cdk1, we measured the mitochondrial ATP generation as an overall mitochondrial functional output. Although ATP production was considerably higher in p53^+/+^ cells than p53 deficient cells, restoring p53 into HCT116 p53^−/−^ cells completely rescued the ATP production. However, wild-type or S315D mutant p53 appeared to have higher ATP production in comparison with p53 deficient or mutant p53 S315A expressing cells (Figure 4A). The expression levels of p53 in HCT116 cells with p53^+/+^, p53^−/−^, p53WT, p53-S315A and p53-S315D mutants were confirmed by western blots (Figure S3A). The presence of p53 is critical for mitochondrial generation of ATP, as it transcriptionally regulates a group of mitochondrial respiration genes [17], [44], [45] and plays a direct role in mitochondrial ATP machinery [46]. Moreover, we also observed that mitochondrial ATP production was induced by the radiation stimulus, possibly due to the mitochondrial influx of proteins (Figure S3B).

We further examined the importance of Ser-315 p53 phosphorylation by cyclin B1/Cdk1 by inhibiting cyclin B1 and Cdk1 with siRNA. Results indicated that the siRNAs against cyclin B1 or Cdk1 were effective in reducing mitochondrial ATP production in HCT116 p53^+/+^ and HCT116 p53^−/−^ cells expressing wild-type p53, approximately 30%; while blocking cyclin B1 or Cdk1 in the p53 deficient or mutant p53 S315A transfectants resulted in merely 5 and 15% reduction in ATP production, respectively (Figure 4B). Therefore, we rationalized that p53 and its Ser-315 phosphorylation is essential for mitochondrial ATP production. Consistent with these results, ATP production was strikingly increased in HCT116 p53^+/+^ and the wide-type p53 transfectants of HCT116 p53^−/−^ that were additionally transfected with mitochondria-targeted cyclin B1/Cdk1 while little ATP enhancement was detected in the p53 deficient or mutant p53 transfectants (Figure 4C). The p53-related mitochondrial ATP production appeared unrelated to the status of mitochondrial biogenesis since no difference in expression of mitochondrial fusion and fission proteins, GTPase, Mfn1 and Drp1, was observed (Figure S4). Thus, we conclude that the enhanced mitochondrial ATP production was due to the p53 Ser-315 phosphorylation by mitochondrial cyclin B1/Cdk1.

### Cyclin B1/Cdk1-mediated mitochondrial p53 Ser-315 phosphorylation maintained mitochondrial membrane potential and cell viability

**Figure 5:**
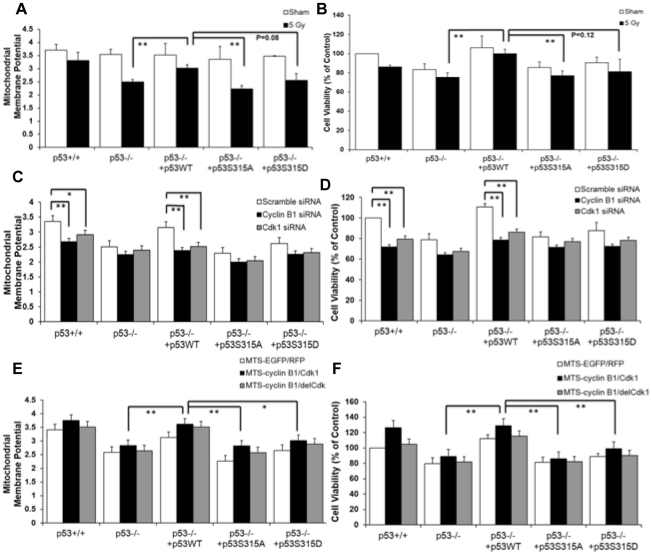
Cyclin B1/Cdk1-mediated p53 phosphorylation enhanced mitochondrial membrane potential and cell viability. Mitochondrial membrane potential (**A**) and cell viability (**B**) were measured in irradiated HCT116 p53^+/+^, p53^−/−^, or HCT116 p53^−/−^ cells stably transfected with wild-type p53, or S315A/S315D mutants (n = 3; mean ± SEM; **P<0.01). Mitochondrial membrane potential (**C**) and cell viability (**D**) were measured in the same set of cells additionally transfected with cyclin B1 or Cdk1 siRNAs for 24 h (n = 3; mean ± SEM; *P<0.05; **P<0.01). Mitochondrial membrane potential (**E**) and cell viability (**F**) were measured in the same set of cells additionally co-transfected with MTS-EGFP and MTS-RFP, or MTS-cyclin B1 and MTS-Cdk1, or MTS-cyclin B1 and MTS-delCdk1 (n = 3; mean ± SEM; *P<0.05; **P<0.01).

Next, we addressed the question of whether mitochondrial membrane potential (Δψ_m_) and cell viability are influenced by the status of cyclin B1/Cdk1-mediated mitochondrial p53 phosphorylation. Compared to the basal Δψ_m_, cells with wild-type p53 (HCT116 p53^+/+^ or HCT116 p53^−/−^ rescued with p53 wild-type) retained a higher Δψ_m_ than HCT116 p53^−/−^ cells and the HCT116 p53^−/−^ cells expressing mutant p53 after irradiation (Figure 5A). Consistent with these results, cells with wild-type p53 also appeared higher cell viability as measured by MTT reduction than the cells with deficient p53 or mutant p53 after treated by either sham or 5 Gy radiation (Figure 5B). Moreover, Cells with wild type p53 status have significantly higher survival determined by clonogenic survival assay after 5 Gy irradiation, whereas cells with p53 deficient or Alanine mutated Ser-315 only had 40% survival rate compared to p53 wild type expressing cells (Figure S5A). These results suggest that p53 may not only be able to protect mitochondria but also provide long term survival for cells against radiation insult.

Compared to scrambled siRNA, pretreatment of cells with siRNAs against cyclin B1 or Cdk1 before irradiation significantly reduced mitochondrial membrane potential and cell viability in cells with wild-type p53, but had minimal effects on cells with deficient or mutated p53 (Figures 5C and D). Further studies with co-transfection of mitochondria-targeted cyclin B1/Cdk1 revealed that radiation-induced reduction of Δψ_m_ and cell viability was nominal in p53 wild-type expressing cells. In contrast, the overexpression of MTS-cyclin B1/Cdk1 had slight effects on both mitochondrial membrane potential and cell viability in cells containing deficient or mutated p53 post-irradiation (Figures 5E and F). These results demonstrate that Ser-315 phosphorylation on p53 by cyclin B1/Cdk1 in mitochondria is essential for maintaining mitochondrial membrane potential and cell viability under the stress of ionizing radiation.

### Cyclin B1/Cdk1-induced p53 phosphorylation inhibited mitochondria-mediated apoptosis

**Figure 6:**
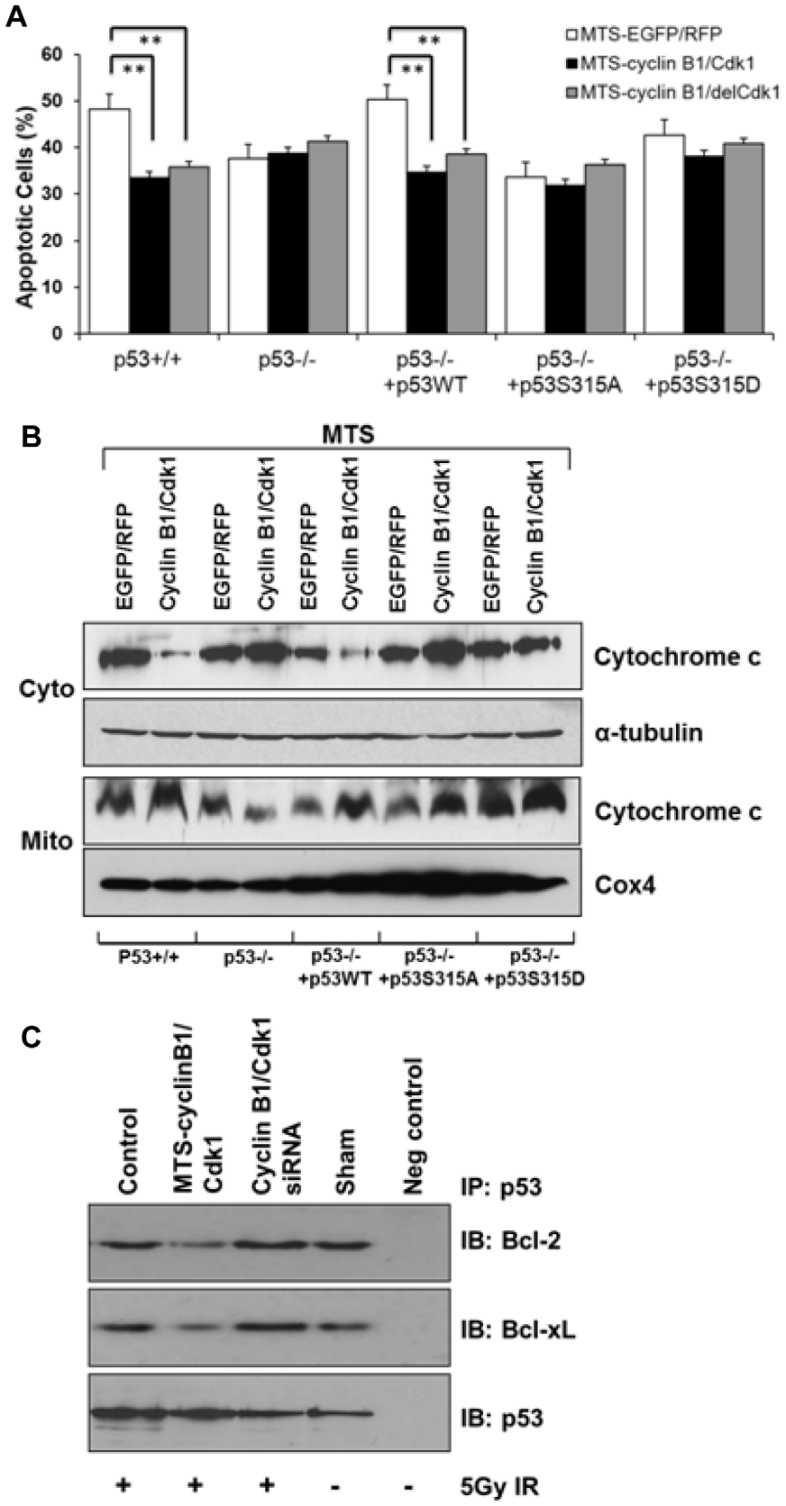
Cyclin B1/Cdk1-mediated p53 phosphorylation inhibited mitochondria-mediated apoptosis. (**A**) HCT116 p53^+/+^, HCT116 p53^−/−^ and HCT116 p53^−/−^ transfectants of wild-type, or mutant S315A, S315D p53 were co-transfected with MTS-EGFP and MTS-RFP, MTS-cyclin B1 and MTS-Cdk1, or MTS-cyclin B1 and MTS-delCdk1 for 24 h and irradiated with 5 Gy. Apoptotic cells with sub G_0_/G_1_ DNA content were measured 24 h post-irradiation by flow cytometry (n = 3; mean ± SEM; **P<0.01). (**B**) Cytosolic and mitochondrial cytochrome c in the same set of cells was measured 24 h after 5 Gy irradiation (α-tubulin and Cox4 included as loading controls). (**C**) Cyclin B1/Cdk1-mediated p53 phosphorylation inhibited the interaction of p53 to Bcl2 or Bcl-xL p53. HCT116 p53^+/+^ cells transfected with MTS-cyclin B1/MTS-Cdk1 or siRNAs against cyclin B1/Cdk1 for 24 h followed by 5 Gy irradiation. Cell lysates were immunoprecipitated with p53 antibody (IP) followed by immunoblotted (IB) using Bcl-2, Bcl-xL or p53 antibodies. HCT116 p53^−/−^ cell lysates were included as negative control.

To determine the potential effect of cyclin B1/Cdk1-phosphorylated p53 in mitochondria-mediated apoptosis, we measured the apoptosis in irradiated HCT116 p53^+/+^ and HCT116 p53^−/−^ cells with different p53 status. Little differences of the radiation-induced apoptosis were detected among HCT116 cells regardless of their p53 status (Figure S5B), which agreed with the previous report [47]. However, inhibition of cyclin B1 by siRNA knockdown along with irradiation dramatically increased the ratio of apoptotic population in cells with wild-type p53, but not in the cells with mutant p53 (Figure S5C). These results suggest that mitochondrial cyclin B1/Cdk1 is required for anti-apoptotic response mediated by p53. Co-overexpression of mitochondrial cyclin B1/Cdk1 also significantly decreased the apoptotic ratio by approximately 20% in cells expressing wild-type p53 after exposure to radiation (Figure 6A). Since initiation of mitochondria-mediated apoptosis starts with permeabilization of the mitochondrial outer membrane and the release of mitochondrial cytochrome c that triggers subsequent activation of caspase cascades [48], we measured levels of cytosolic and mitochondrial cytochrome c and observed a strikingly less cytosolic cytochrome c released from mitochondria after irradiation in cells expressing wild-type p53 transfected with MTS-cyclin B1/MTS-Cdk1, but not in cells with deficient or mutant p53 (Figure 6B).

In mitochondria-mediated pathways, p53 is reported to play an apoptotic role through disrupting the function of mitochondrial-membrane associated Bcl-2 and Bcl-xL [49], [50]. We then tested whether the cyclinB1/Cdk1-phosphorylated p53 interrupts its association with Bcl-2 or Bcl-xL. Results shown in Figure 6C indicated that levels of Bcl-2 or Bcl-xL protein pulled down by anti-p53 were reduced in irradiated cells transfected with MTS-cyclin B1/Cdk1, which was noticeably increased by transfection with siRNAs against cyclin B1 or Cdk1 (Figure 6C). These findings suggest a new important link between cyclin B1/Cdk1-mediated p53 phosphorylation and mitochondrial apoptosis, in which the phosphorylation was able to lead to reduced association between p53/Bcl-2 and p53/Bcl-xL, followed by inhibition of mitochondria-mediated apoptosis. Overall, we conclude that the mitochondrial cyclin B1/Cdk1-induced p53 phosphorylation functions as an anti-apoptotic event via increasing mitochondrial ATP production, maintaining mitochondrial membrane potential, and interrupting the p53/Bcl-2 and p53/Bcl-xL association.

## Discussion

Under different stress conditions, the tumor suppressor p53 is shown to translocate to mitochondria [4], [51] to induce pro- or anti-apoptotic responses depending on its phosphorylation status [8], [10], [15], [52]. Although the pathways accounting for p53 regulated pro-apoptotic functions are well defined [53], the anti-apoptotic response induced by mitochondrial p53 is largely unknown. In this report, using p53 wild type and deficient tumor cells, we provide the circumstantial evidence suggesting a novel pro-survival regulation of mitochondrial p53 Ser-315 phosphorylation by cyclin B1/Cdk1 that are also translocated to mitochondria. In response to ionizing radiation, not only the total protein levels of p53 and cyclin B1/Cdk1 are enhanced but also a substantial amount of each protein is increased in mitochondria. The mitochondrial cyclin B1/Cdk1 is found to phosphorylate p53 at Ser-315 residues, causing an increased mitochondrial integrity with enhanced ATP production and mitochondrial membrane potential and reduced apoptosis.

Approximately 50 percent of human cancers contain different p53 mutations [54], [55], [56]. It has been faithfully believed that the major function of p53 is to induce apoptosis and the defects in p53 apoptotic function lead to the possibility of cell transformation and tumor-growth advantages [57], [58]. However, recent reports suggest that p53 may provide protective functions for endothelial cell of gastrointestinal track against radiation [10]. Additional evidences indicate that p53 mutations in two human sarcoma cell lines are linked with their radiosensitivity [59], and the presence of p53 in mitochondria appears not to be associated with mitochondrial apoptosis [11], [47]. Our evidence present that mitochondrial p53 acts as an advocate of mitochondrial integrity with anti-apoptotic function suggesting a complex regulation on mitochondrial p53 and apoptosis. The mechanism of such pro-survival function of mitochondrial p53 is linked with the kinase activity of cyclin B1/Cdk1 that also translocate to mitochondria upon genotoxic stress. Inhibition of cyclin B1/Cdk1 by siRNA resulted in reduction of cell viability, mitochondrial membrane potential that led to increased apoptotic cell numbers. Co-overexpression of the intact mitochondrial cyclin B1/Cdk1 resulted in the opposite phenotypes. In contrast, the presence or absence of cyclin B1/Cdk1 in mitochondria showed little effect in radiosensitivity of cells expressing deficient p53 or Ser-315 mutated p53. In the light of the recent study reporting that inhibition of Cdk1 leads to p53-mediated apoptosis [60], our data extend the current knowledge with mechanistic insights that phosphorylation of mitochondrial p53 by cyclin B1/Cdk1 preserves mitochondrial integrity and suppresses mitochondria-mediated apoptosis.

The cyclin B1/Cdk1-mediated Ser-315 phosphorylation is found to sequester p53 from binding to anti-apoptotic Bcl-2 and Bcl-xL, which are the major anti-apoptotic proteins located in mitochondria [61], [62]. Based on these findings, it is clear that p53 Ser-315 is phosphorylated by mitochondrial cyclin B1/Cdk1 complex after it enters mitochondria. However, our current data cannot exclude the possibility that the phosphorylated p53 by cyclin B1/Cdk1 in mitochondria may be able to migrate to cytoplasm and inhibit the extrinsic pathways towards apoptosis. We, therefore, conclude that the improvement of overall mitochondrial integrity and ATP production correlated with the inhibited mitochondrial extrinsic apoptosis may contribute to a significant portion of resistance to genotoxic stress. With these results in mind together with the proposed pro-survival functions of p53 [63], we propose that the specific localization and phosphorylation status of p53 in mitochondria should be considered as a major factor in deciding anti-apoptotic response.

This study has presented evidences showing irradiation, at the moderate dose of 5 Gy, induced mitochondrial influx of p53, cyclin B1 and Cdk1, but does not include the question of which mechanisms causing the translocation of these nuclear proteins to mitochondria. There is a possibility that elevated levels of cellular and mitochondrial ROS or mitochondrial DNA damage induced by radiation is responsible for such protein translocation. However, nuclear DNA damage, a prominent feature of irradiation stress in mammalian cells, could serve as the key factor triggering such subcellular protein traffic. Therefore, our study strongly suggests that the mitochondrial protein influx after genotoxic radiation is initiated by the nuclear DNA damage. Further studies are needed to elucidate the detailed mechanisms accounting for the translocation of proteins from nucleus to mitochondria.

**Figure 7:**
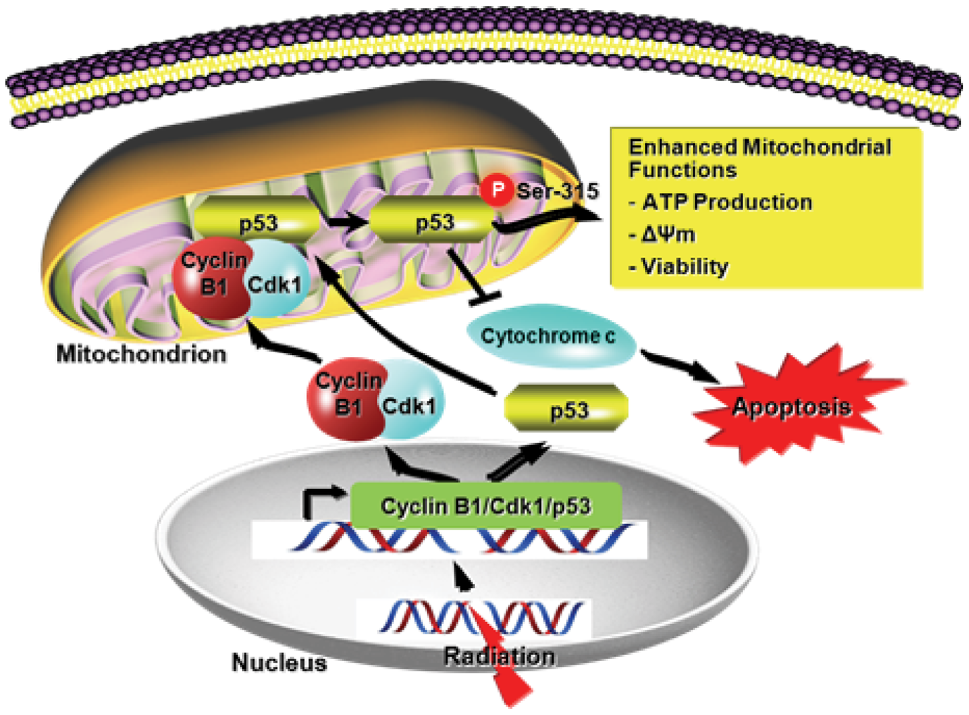
Schematic illustration of the nucleus-to-mitochondria signaling under DNA-damaging stress. DNA-damage irradiation stimulates the gene expression and mitochondrial translocation of p53, cyclin B1 and Cdk1. Mitochondrial cyclin B1 and Cdk1 form a functional complex to phosphorylate p53, which leads to enhanced ATP production and suppression of mitochondria-mediated apoptosis.

Multiple kinases and phosphatases in mammals are identified to translocate to mitochondria and play critical roles in modulating mitochondrial function [64]. We show here that irradiation not only stimulated the expression of cyclin B1 and Cdk1, but also promoted their translocation to mitochondria where the p53 Ser-315 is phosphorylated by Cdk1 that apparently formed the active complex with its partner cyclin B1 in the mitochondria. Although p53 phosphorylation by cyclin B1/Cdk1 has been previously described [36] and p53 phosphorylation does not induce its mitochondrial translocation under stress [65], our current study suggests a novel feature of cyclin B1/Cdk1 that phosphorylates p53 within mitochondria. In this case, accumulation of nuclear DNA damage induced by irradiation reaching a certain threshold is able to trigger adaptive cellular response by activating a nuclear-to-mitochondrial protein influx including p53, cyclin B1 and Cdk1. Such mitochondria-targeted protein influx under a critical DNA damage condition appears to quickly enhance mitochondrial functions in order to supply sufficient cellular energy demand required for DNA damage repair. However, in p53 deficient or Ser 315-mutated p53 expressing cells, such radioadaptive response is absent possibly due to the lack of cyclin B1/Cdk1-mediated mitochondrial p53 phosphorylation and a consequential improvement of mitochondrial function. Our data also suggest that restoration of wild-type p53 along with mitochondria-targeted cyclin B1/Cdk1 reinstated their mitochondrial function. Further characterization of mitochondrial function in p53 mutant versus p53 wild type tumor cells under geno- and cytotoxic conditions will generate important insights to explain the different response of cancer patients undergoing anti-cancer therapy. Combined this novel report with previously published results, we propose a mitochondria-mediated adaptive radioprotection of tumor cells with intact p53 status. As illustrated in Figure 7, mitochondrial translocation of p53 and cyclin B1/Cdk1 is activated under acute DNA damage response. The phosphorylation of mitochondrial p53 by cyclin B1/Cdk1 stabilizes mitochondrial membrane potential and enhances mitochondrial ATP production, sequentially supplies the ATP required to repair damaged DNA and other critical cellular elements. Our model of mitochondrial p53 phosphorylation by cyclin B1/Cdk1 signaling cascade provides an important approach to further elucidate the mechanism underlying therapy-resistant phenotype of tumor cells against genotoxic stress.

In conclusion, the DNA damaging anti-cancer agents not only can induce the gene expression of p53 and cyclin B1/Cdk1 but also enhance their translocation to mitochondria. The subsequent phosphorylation of p53 Ser-315 by mitochondria-translocated cyclin B1/Cdk1 appears to be an important step in enhancing mitochondrial integrity with increased mitochondrial ATP production and reduced mitochondrial apoptosis for tumor radioresistance. Targeting such nuclear-to-mitochondrial protein traffic may have a profound implication in improving the efficacy of tumor radiotherapy.

## Materials and Methods

### Cell culture and radiation exposure

Human colon carcinoma cell lines HCT116 p53^+/+^ and HCT116 p53^−/−^ were kindly provided by Dr. Bert Vogelstein (Johns Hopkins University, MD) and maintained in McCoy's 5A medium supplemented with 10% fetal bovine serum (HyClone, Logan, UT), penicillin (100 units per ml) and streptomycin (100 µg/ml) in a humidified incubator at 37°C (5% CO2). Exponentially growing cells in T75 flask with 70–80% confluence were exposed to radiation at room temperature using a Cabinet X-rays System Faxitron Series (dose rate: 0.997 Gy/min; 130 kVp; Hewlett Packard, McMinnville, OR). Cells sheltered from radiation were used as the sham-IR control.

### Cell transfection

Scrambled siRNA was purchased from Ambion (Austin, TX) (4635). siRNA targeting cyclin B1 or Cdk1 mRNAs was designed and synthesized with the *Silencer* siRNA Construction Kit (Ambion). The primers used to synthesize the siRNAs were as follows:

cyclin B1, 5′ AATTTCTGGAGGGTACATTTCCCTGTCTC 3′ (Sense) and

5′ AAGAAATGTACCCTCCAGAAACCTGTCTC 3′ (Antisense);

Cdk1, 5′ AAGGGGTTCCTAGTACTGCAACCTGTCTC 3′ (Sense) and

5′ AATTGCAGTACTAGGAACCCCCCTGTCTC 3′ (Antisense).

Cells were seeded to achieve 30%–50% confluence on the day of transfection. Transient transfection of siRNA was performed using Lipofectamine™ RNAiMAX reagent (Invitrogen, Carlsbad, CA). In brief, cells were seeded in 35 mm plates and cultured in antibiotic-free medium for 24 h, and then were transfected with 20 nM of siRNAs. Scrambled RNA Duplex (Ambion) served as the control. All transfectants were maintained in antibiotic-free complete medium.

### Mitochondrial fractionation

Exponentially growing HCT116 p53^+/+^ cells at 50–80% confluence were used for protein fractionation. Cytosolic and mitochondrial fractions were collected using mitochondria isolation kit from Thermo Scientific (Rockford, IL) (89874). Briefly, cells were incubated in ice-cold hypotonic buffer containing 10mM NaCl, 1.5 mM MgCl2 and 10mM Tris-HCl pH 7.5 for 20 min and cell membrane was disrupted by glass pestle and buffer containing 2M sucrose, 35 mM EDTA and 50 mM Tris-HCl pH 7.5 was added to prevent damages to mitochondria by osmotic pressure. Mitochondrial fraction was then separated by centrifugation at 12000 rpm for 20 min.

### Immunoblotting and Co-immunoprecipitation

Unless noted otherwise, cytosolic fraction or total cell or mitochondrial lysates (20 µg) were separated by SDS-PAGE and blotted onto nitrocellulose membrane. The membrane was incubated with specific primary antibody overnight at 4°C, followed by the horseradish peroxidase-conjugated secondary antibody for 1h, and visualized by the ECL Western blotting detection system (Amersham, Arlington Heights, IL). The antibody against p53 (sc-1019), actin (sc-8432), cyclin B1 (sc-245), p53 (sc-126), Mfn1 (sc-166644) and Drp1 (sc-32898) were purchased from Santa Cruz Biotechnology (Santa Cruz, CA). Anti-Cox4 (ab14744) was bought from Abcam (Cambridge, MA). The secondary antibodies were from ebioscience (San Diego, CA) (18–8816 and 18–8817).

Mitochondrial extracts were prepared with lysis buffer containing 10 mM HEPES, 10 mM KCl, 1.5 mM MgCl2, 0.5 mM DTT, 1% NP40, 1 mM phenylmethylsulfonyl fluoride, 25% glycerol, and 0.2 mM EDTA. Extracts were further centrifuged at 4°C for 15 min and precleared by 1-h treatment with prebleed sera or normal mouse or rabbit immunoglobulin G and 20 µL of 1∶1 slurry of protein A/G-Sepharose beads at 4°C. Immunoprecipitation preceded at least 2 h at 4°C, followed by the addition of protein A/G beads and further 30 min incubation. Beads were collected by brief centrifugation, washed four times with 1× PBS washing buffer, boiled in denature loading buffer, separated by 10% SDS-PAGE gel, and followed by immunoblotting as stated above.

### Plasmid Construction

pEGFP-N1 vector from Clontech (Mountain View, CA) (Accession # U55762) was used to insert the mitochondrial targeting sequence from Cox8a following by cyclin B1 or Cdk1 gene. In the case of RFP, EGFP is replaced by RFP at the restriction sites indicated in the plasmid sequences.

### Fluorescence microscopy analyses

HCT116 p53^+/+^ cells were transfected with vectors containing EGFP or RFP. For Mitotracker staining, 5 µM Mitotracker Red or Green (Invitrogen) were added into cell medium for 5 min and rinse twice with PBS then fixed in ice-cold 3.7% formaldehyde for fluorescence microscopy.

### *In vivo* kinase assay

Mitochondrial Cdk1-cyclin B1 complexes were purified by immunoprecipitation from 2.4 mg of extract derived from mitochondrial fraction of irradiated cells using 1.2 µg of mouse monoclonal anti-Cdk1 antibody and 20 µl of protein G-Sepharose beads. After a 3-h incubation at 4°C, the beads were washed three times with 1% Triton-X 100 buffer and two times with kinase buffer (50 mM Tris-HCl pH 7.5, 10 mM MgCl_2_, 1 mM EGTA, 2 mM DTT and 0.01% Brij 35. 8 µg of GST or GST-p53 substrate was added in kinase buffer containing 1 µl of [γ-^32^P] ATP (10 µCi/µl) (Perkin Elmer) and 0.1 M cold ATP. The reaction was incubated at 30°C for 30 min and stopped by boiling in 5× LSB for 5 min. Purified Cdc2 (Cdk1-cyclin B complex) kinase was purchased from New England Biolabs (Ipswich, MA) and used according to the manufacturer's instructions.

### Measurement of ATP production in mitochondria

To measure ATP production, ATP was extracted from cells with 2.5% trichloroacetic acid then neutralized with Tris pH 7.75. The ATP extracts were used to measure ATP production using ATP determination kit (Invitrogen) according to the manufacturer's instructions, with luminometer (Turner Biosystem, Sunnyvale, CA). The experiments were performed three times individually and each experiment contained triple replicates.

### Measurement of mitochondrial membrane potential

Mitochondrial membrane potential measurement was performed as previously described [66]. Briefly, mitochondrial membrane potential was measured using a fluorescent cationic dye, 5,5′,6,6′-tetrachloro-1,1′,3,3′-tetraethyl-benzamidazolocarbocyanin iodide (JC-1; Molecular Probes, Eugene, OR, USA). The changes in membrane potential were determined by the levels of relative fluorescence units, using a SpectraMax M5 MultiMode Microplate Reader (Molecular Devices, Sunnyvale, CA, USA), with a 485 nm excitation filter and a 525–595 nm emission filter.

### Cell viability assay

Exponentially growing cells were plated at cell densities of 10,000 cells per well in 96-well tissue culture plates for 24 h. Cells were then exposed to sham or 5 Gy X-rays. 24 h post-irradiation, cell proliferation was measured by MTT assay according to the manufacturer's instructions (Promega, Madison, WI). Briefly, 10 µl of MTT reagent (5 mg/ml in water) was added into each well and incubated for 2 h. Then 100 µl of 10% SDS with 0.01M HCl was added into each well and incubated for an additional 2 h. The plates were read with micro-plate reader at absorbance of 570 nm. The experiments were independently performed three times and each experiment contained triple replicates.

### FACS analysis

To detect apoptotic cells, exponentially growing cells were exposed to 5 Gy of X-rays. At 24 h post-irradiation, the cells were trypsinized and fixed with ice-cold 70% (v/v) ethanol. The cell pellets were prepared by centrifugation at 200 g for 5 min, washed with phosphate-buffered saline (pH 7.4), and resuspended in phosphate buffer saline containing propidium iodide (50 µg/ml), 0.1 mg/ml bovine serum albumin, Triton X-100 (0.1%, v/v), and DNase-free RNase (0.2 mg/ml) for 30 min at room temperature. DNA content was determined by flow cytometry analysis using a FACScan flow cytometer (Becton Dickinson, San Jose, CA).

### Statistical analysis

The data are presented as the means ± S.E.M. Statistical significance among groups was determined by using paired, two-tailed Student's *t* tests with SAS software (version 9). The findings were considered significant at P<0.05 and highly significant at P<0.01.

## Supporting Information

Figure S1 Mitochondrial interaction between p53 and Cdk1. (A) Cdk1 and p53 were co-immunoprecipitated from mitochondrial fractions of HCT116 p53+/+ exposed to sham or 5 Gy X-rays at 2 and 4 h post-irradiation. Whole cell lysate of HCT116 p53−/− was used as the negative control. (B) Synthesis and purification of GST-tagged p53 proteins. GST-tagged p53 wild-type and mutants (S315A and S315D) were synthesized in BL-21 E.Coli and purified by immobilized gluthathione beads (Thermo Scientific).
(0.28 MB TIF)

Figure S2 Mitochondrial cyclin B1/Cdk1 phosphorylated Ser-315 residue of p53. (A) Phosphorylated p53 at Ser-315 was detected by immunoblotting from whole cell lysate of HCT116 cells exposed to 10 Gy X-rays at indicated time. Phosphorylation of p53 at Ser-315 was enhanced by ionizing radiation. (B) Fluorescence immunostaining of 5 Gy irradiated mouse xenograft tissue generated from human glioblastoma cell line U87 showing mitochondria localization of phosphorylated p53 (Ser-315) and Cdk1. Tissue were collected 24 h after irradiation. Cox4 staining served as the mitochondrial marker (scale bar = 5 µm). (C) Expression of cyclin B1 and Cdk1 were detected by immunoblotting in whole cell lysates of HCT116 cells transfected with cyclin B1, Cdk1 and scrambled siRNA at the concentration of 20 nM, cells transfected with Lipofectamine only were included as control. (D) Mitochondrial cyclin B1, cyclin B1-EGFP, Cdk1 and Cdk1-RFP were detected by immunoblotting in HCT116 cells transfected with mitochondria-targeted EGFP and RFP (MTS-EGFP/RFP; served as vector control), mitochondria-targeted cyclin B1-EGFP and Cdk1-RFP (MTS-cyclin B1/MTS-Cdk1), and mitochondria-targeted cyclin B1-EGFP and Cdk1 with deletion in its catalytic loop (residue 122–132) linked with RFP (MTS-cyclin B1/MTS-delCdk1) along with mitochondrial fraction of HCT116 cells without any transfection. (E) Representative images of HCT116 p53+/+ cells transfected with MTS-EGFP and MTS-RFP with Mitotracker for mitochondrial staining (scale bar = 5 µm).
(2.95 MB TIF)

Figure S3 Effect of p53 in mitochondrial ATP production. (A) HCT116 p53−/− cells were transfected with p53 (wild-type) and mutants (S315A and S315D) linked with myc-tag in plasmid containing Zeocin resistance gene and the transfectants were selected with Zeocin (150 µg/ml) for 6 days. Expression of p53 in the stable transfectants were detected by western blot comparing to p53 expression in HCT116 p53+/+ and HCT116 p53−/−. (B) ATP production was measured in HCT116 p53+/+ and HCT116 p53−/− cells exposed to sham or 5 Gy X-rays at indicated time points after irradiation. Displayed is the mean ±SEM; n = 3; **P<0.01. The enhanced ATP production is likely due to the influx of cyclin B1, Cdk1 and p53 to mitochondria.
(0.37 MB TIF)

Figure S4 Limited effect on mitochondrial fission by ionizing radiation and mitochondrial cyclin B1, Cdk1 and p53. Expression of Drp1 and Mfn1 was detected by immunoblotting from 5 Gy irradiated whole cell and mitochondrial fraction of HCT116 p53+/+ cells at indicated time points post-irradiation. β-actin and Cox4 served as the loading controls.
(0.31 MB TIF)

Figure S5 Pro-survival effects of cyclin B1/Cdk1-mediated p53 phosphorylation. (A) Clonogenic survival of HCT116 p53+/+, HCT116 p53−/−, HCT116 p53−/− +p53WT, HCT116 p53−/− +p53S315A and HCT116 p53−/− +p53S315D cells exposed to 5 Gy X-rays, colonies were stained and counted 10 days post-irradiation (n = 3, mean ± SEM; **P<0.01). (B) Apoptotic cell measurement by flow cytometry after 5 Gy irradiation. Cell were collected at 24 h after irradiation, fixed and stained by propidium iodine for apoptotic cell counts (n = 3, mean ± SEM). (B) The same set of cell lines were transfected with scrambled, cyclin B1 or Cdk1 siRNA for 24 h and irradiated with 5 Gy X-rays. Cells were collected at 24 h post-irradiation and analyzed by flow cytometry with propidium iodine staining (n = 3, mean ± SEM; *P<0.05; **P<0.01).
(0.62 MB TIF)
