# Expression of Concern: Cyclin B1/Cdk1 Phosphorylation of Mitochondrial p53 Induces Anti-Apoptotic Response

**DOI:** 10.1371/journal.pone.0220519

**Published:** 2019-07-25

**Authors:** The PLOS ONE Editors

After the publication of [1], a concern was raised that the p53 HCT116 p53^-/-^ panel of Fig 1C appears similar to the p53 HK-18 panel of Fig 1D when rotated and vertically stretched.

The authors explained that due to an error during figure preparation, the images had been duplicated in Fig 1C and Fig 1D. They were able to provide the uncropped image underlying Fig 1C p53 panel but could not locate the blot for Fig 1D p53 panel owing to the length of time since the experiments were carried out.

In light of the data unavailability and unresolved concern about Fig 1D, the *PLOS ONE* Editors post this Expression of Concern.
