# Supplementary figures and images for: Cyclin B1/Cdk1 Phosphorylation of Mitochondrial p53 Induces Anti-Apoptotic Response
Source: PLoS One. 2010 Aug 23;5(8):e12341. doi: 10.1371/journal.pone.0012341 (PMC2925892; doi:10.1371/journal.pone.0012341)

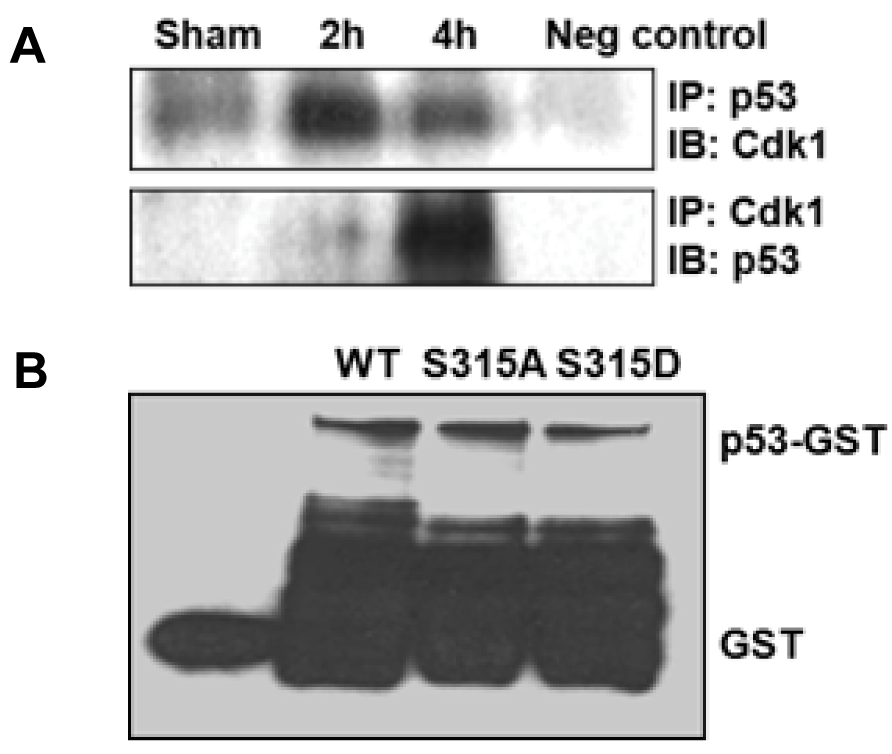

Supplement: Figure S1 — Mitochondrial interaction between p53 and Cdk1. (A) Cdk1 and p53 were co-immunoprecipitated from mitochondrial fractions of HCT116 p53+/+ exposed to sham or 5 Gy X-rays at 2 and 4 h post-irradiation. Whole cell lysate of HCT116 p53−/− was used as the negative control. (B) Synthesis and purification of GST-tagged p53 proteins. GST-tagged p53 wild-type and mutants (S315A and S315D) were synthesized in BL-21 E.Coli and purified by immobilized gluthathione beads (Thermo Scientific). (0.28 MB TIF) [file pone.0012341.s001.tif]

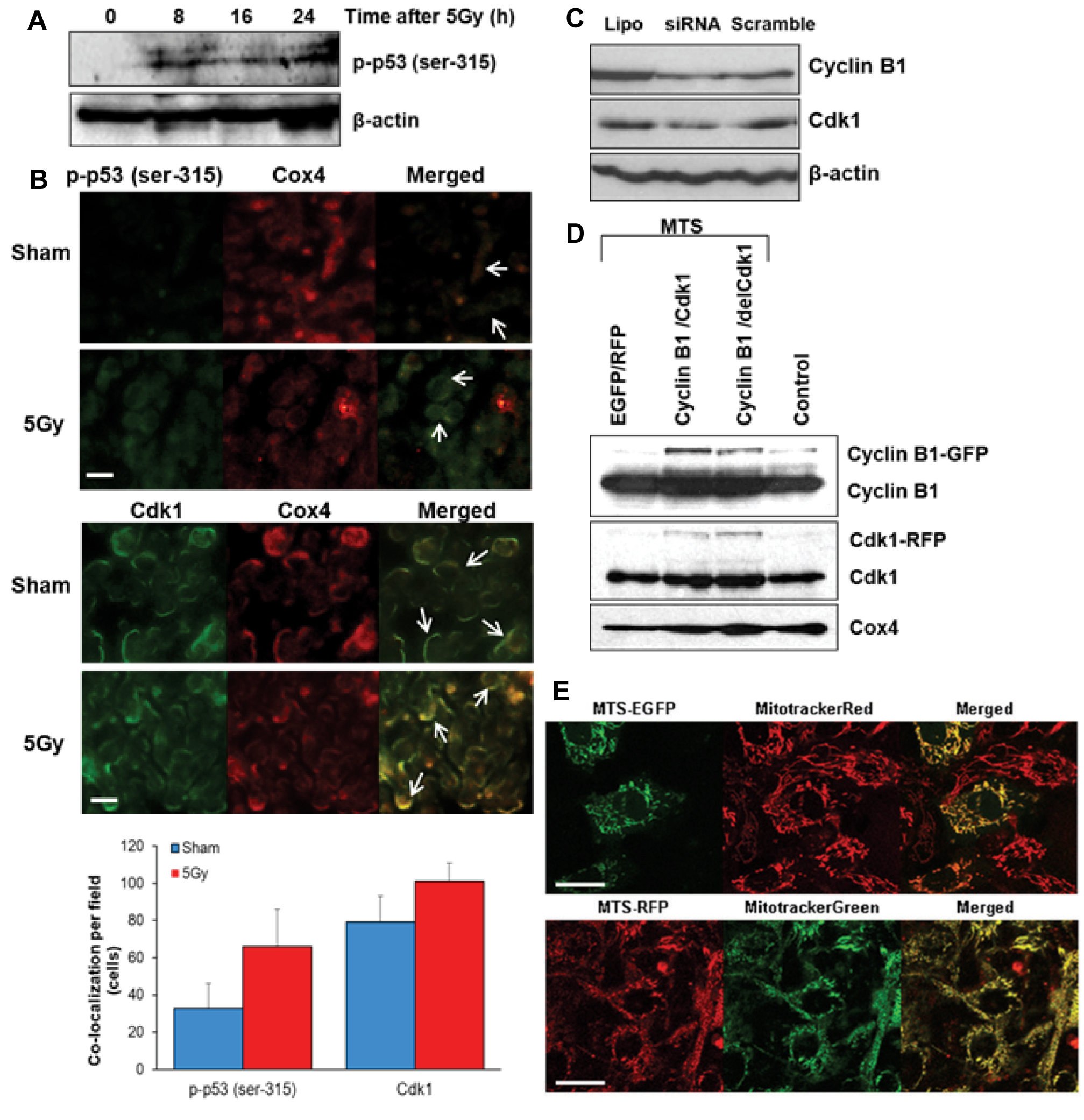

Supplement: Figure S2 — Mitochondrial cyclin B1/Cdk1 phosphorylated Ser-315 residue of p53. (A) Phosphorylated p53 at Ser-315 was detected by immunoblotting from whole cell lysate of HCT116 cells exposed to 10 Gy X-rays at indicated time. Phosphorylation of p53 at Ser-315 was enhanced by ionizing radiation. (B) Fluorescence immunostaining of 5 Gy irradiated mouse xenograft tissue generated from human glioblastoma cell line U87 showing mitochondria localization of phosphorylated p53 (Ser-315) and Cdk1. Tissue were collected 24 h after irradiation. Cox4 staining served as the mitochondrial marker (scale bar = 5 µm). (C) Expression of cyclin B1 and Cdk1 were detected by immunoblotting in whole cell lysates of HCT116 cells transfected with cyclin B1, Cdk1 and scrambled siRNA at the concentration of 20 nM, cells transfected with Lipofectamine only were included as control. (D) Mitochondrial cyclin B1, cyclin B1-EGFP, Cdk1 and Cdk1-RFP were detected by immunoblotting in HCT116 cells transfected with mitochondria-targeted EGFP and RFP (MTS-EGFP/RFP; served as vector control), mitochondria-targeted cyclin B1-EGFP and Cdk1-RFP (MTS-cyclin B1/MTS-Cdk1), and mitochondria-targeted cyclin B1-EGFP and Cdk1 with deletion in its catalytic loop (residue 122–132) linked with RFP (MTS-cyclin B1/MTS-delCdk1) along with mitochondrial fraction of HCT116 cells without any transfection. (E) Representative images of HCT116 p53+/+ cells transfected with MTS-EGFP and MTS-RFP with Mitotracker for mitochondrial staining (scale bar = 5 µm). (2.95 MB TIF) [file pone.0012341.s002.tif]

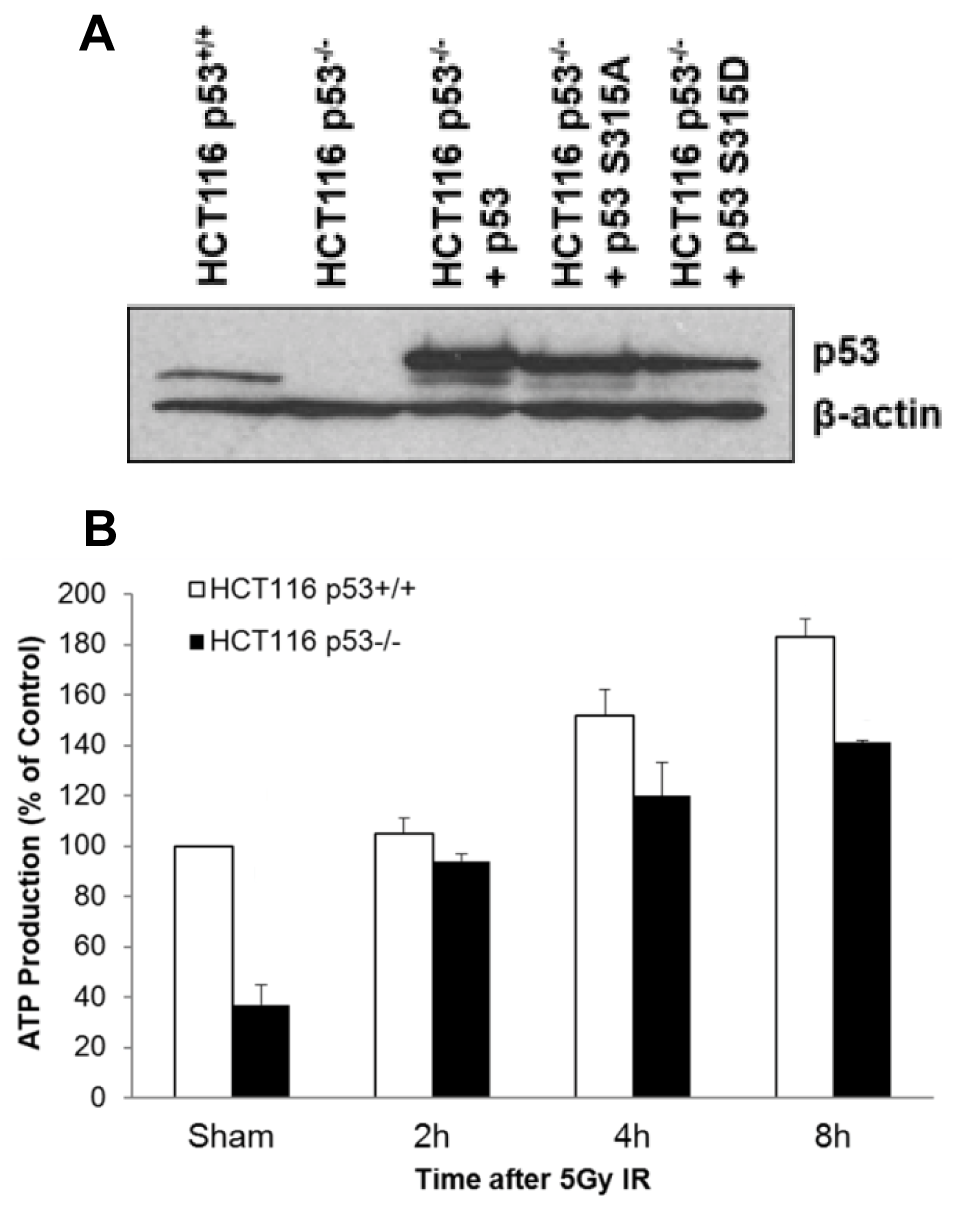

Supplement: Figure S3 — Effect of p53 in mitochondrial ATP production. (A) HCT116 p53−/− cells were transfected with p53 (wild-type) and mutants (S315A and S315D) linked with myc-tag in plasmid containing Zeocin resistance gene and the transfectants were selected with Zeocin (150 µg/ml) for 6 days. Expression of p53 in the stable transfectants were detected by western blot comparing to p53 expression in HCT116 p53+/+ and HCT116 p53−/−. (B) ATP production was measured in HCT116 p53+/+ and HCT116 p53−/− cells exposed to sham or 5 Gy X-rays at indicated time points after irradiation. Displayed is the mean ±SEM; n = 3; **P<0.01. The enhanced ATP production is likely due to the influx of cyclin B1, Cdk1 and p53 to mitochondria. (0.37 MB TIF) [file pone.0012341.s003.tif]

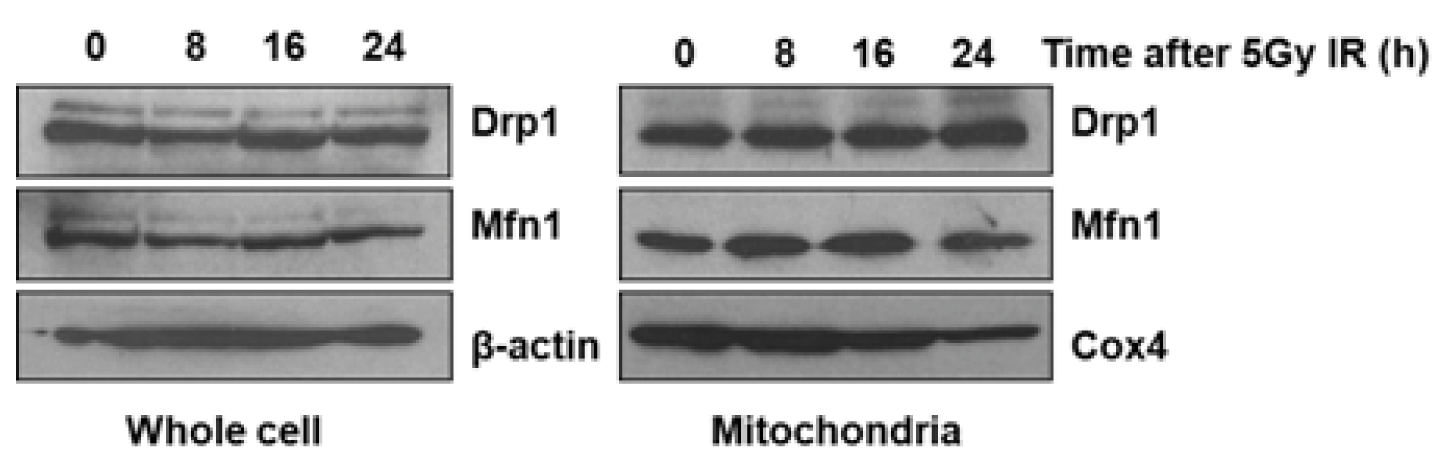

Supplement: Figure S4 — Limited effect on mitochondrial fission by ionizing radiation and mitochondrial cyclin B1, Cdk1 and p53. Expression of Drp1 and Mfn1 was detected by immunoblotting from 5 Gy irradiated whole cell and mitochondrial fraction of HCT116 p53+/+ cells at indicated time points post-irradiation. β-actin and Cox4 served as the loading controls. (0.31 MB TIF) [file pone.0012341.s004.tif]

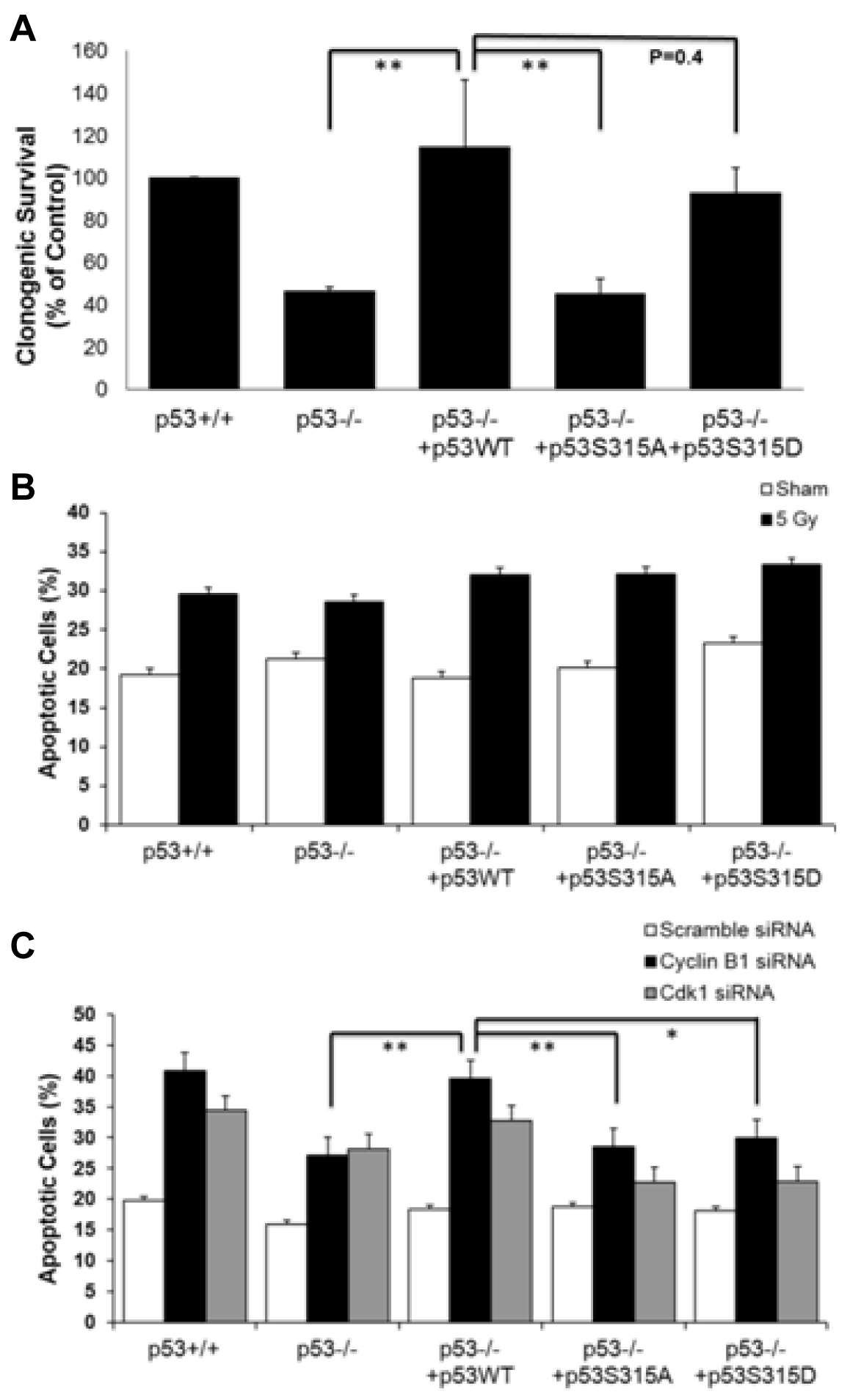

Supplement: Figure S5 — Pro-survival effects of cyclin B1/Cdk1-mediated p53 phosphorylation. (A) Clonogenic survival of HCT116 p53+/+, HCT116 p53−/−, HCT116 p53−/− +p53WT, HCT116 p53−/− +p53S315A and HCT116 p53−/− +p53S315D cells exposed to 5 Gy X-rays, colonies were stained and counted 10 days post-irradiation (n = 3, mean ± SEM; **P<0.01). (B) Apoptotic cell measurement by flow cytometry after 5 Gy irradiation. Cell were collected at 24 h after irradiation, fixed and stained by propidium iodine for apoptotic cell counts (n = 3, mean ± SEM). (B) The same set of cell lines were transfected with scrambled, cyclin B1 or Cdk1 siRNA for 24 h and irradiated with 5 Gy X-rays. Cells were collected at 24 h post-irradiation and analyzed by flow cytometry with propidium iodine staining (n = 3, mean ± SEM; *P<0.05; **P<0.01). (0.62 MB TIF) [file pone.0012341.s005.tif]
